# GABR_A3_ promotes lymphatic metastasis in lung adenocarcinoma by mediating upregulation of matrix metalloproteinases

**DOI:** 10.18632/oncotarget.8700

**Published:** 2016-04-11

**Authors:** Liping Liu, Chenglin Yang, Jianfei Shen, Liyan Huang, Weixuan Lin, Hailing Tang, Wenhua Liang, Wenlong Shao, Haibo Zhang, Jianxing He

**Affiliations:** ^1^ The Translational Medicine Laboratory, State Key Laboratory of Respiratory Disease, The First Affiliated Hospital of Guangzhou Medical University, Guangzhou, China; ^2^ Southern Medical University, Guangzhou, China; ^3^ Department of Thoracic Surgery, State Key Laboratory of Respiratory Disease, The First Affiliated Hospital of Guangzhou Medical University, Guangzhou, China; ^4^ Keenan Research Centre for Biomedical Science of St. Michael's Hospital, Department of Anesthesia, Medicine and Physiology, University of Toronto, Toronto, Ontario, Canada

**Keywords:** non-small cell lung cancer, GABR_A3_, MMP, JNK/AP-1, lymphatic metastasis

## Abstract

Tumor metastasis is the main reason for the poor prognosis of lung cancer patients. The GABAA receptor subunit GABR_A3_ is reportedly upregulated in lung cancer. Herein, we show that high GABR_A3_ protein expression in lung adenocarcinoma correlated positively with disease stage, lymphatic metastasis status and poor patient survival. In addition, GABR_A3_ induced MMP-2 and MMP-9 expression through activation of the JNK/AP-1 signaling pathway, which enhanced lymphatic metastasis by lung adenocarcinoma both *in vitro* and *in vivo*. These results indicate that GABR_A3_ promotes lymph node metastasis and may thus be an effective therapeutic target for anticancer treatment.

## INTRODUCTION

Lung cancer is a common malignancy with a high mortality rate that results in approximately a quarter million deaths per year in the United States alone [1]. Non-small cell lung cancer (NSCLC) accounts for approximately 85% of all cases of lung cancer, more than 40% of which is lung adenocarcinoma (LUAD) [2]. Despite improvements in diagnostics and treatment strategies, the prognosis of metastatic lung adenocarcinoma remains poor, with the average 5-year survival rate of approximately 15% [3]. There is thus need to better understand the mechanisms of tumor metastasis in NSCLC and for development of a pharmacological intervention.

Gamma-aminobutyric acid (GABA) is an inhibitory neurotransmitter in the mammalian brain that specifically interacts with two major classes of receptors: GABA A receptor (α1–6, β1–3, γ1–3, δ, ε, θ, π, ρ1–3) and GABA B receptor (GABBR_1_ and GABBR_2_) [4–6]. Fava et al. [7] reported that GABA treatment decreased the proliferation and metastatic potential of cholangiocarcinoma, and Joseph et al. [8] reported that GABA reduced migration of colon cancer. By contrast, Azuma et al. [9] showed that GABA promotes metastasis and invasion by prostate cancer cells through upregulation of metalloproteinase expression. One explanation for these conflicting results may be that different GABA receptor subunits mediate different responses via distinct intracellular signaling pathways, leading to beneficial or deleterious effects.

We previously investigated GABA receptor expression profiles in samples of NSCLC and non-cancerous lung tissues and found that gene expression of GABR_A3_, GABR_E_ and GABBR_2_ was significantly higher in primary NSCLC tissues [10]. Moreover, expression of GABR_A3_ mRNA was associated with a poor prognosis in patients with NSCLC. Consistent with that observation, Liu et al. found that GABR_A3_ gene is overexpressed in NSCLC [11]. This suggests that dominant expression of the GABR_A3_ subunit may result in cancer progression. To test that idea, we assessed the effect GABR_A3_ on the development of lymphatic metastasis in NSCLC.

## RESULTS

### Upregulation of GABR_A3_ levels correlates with progression of LUAD

**Figure 1:**
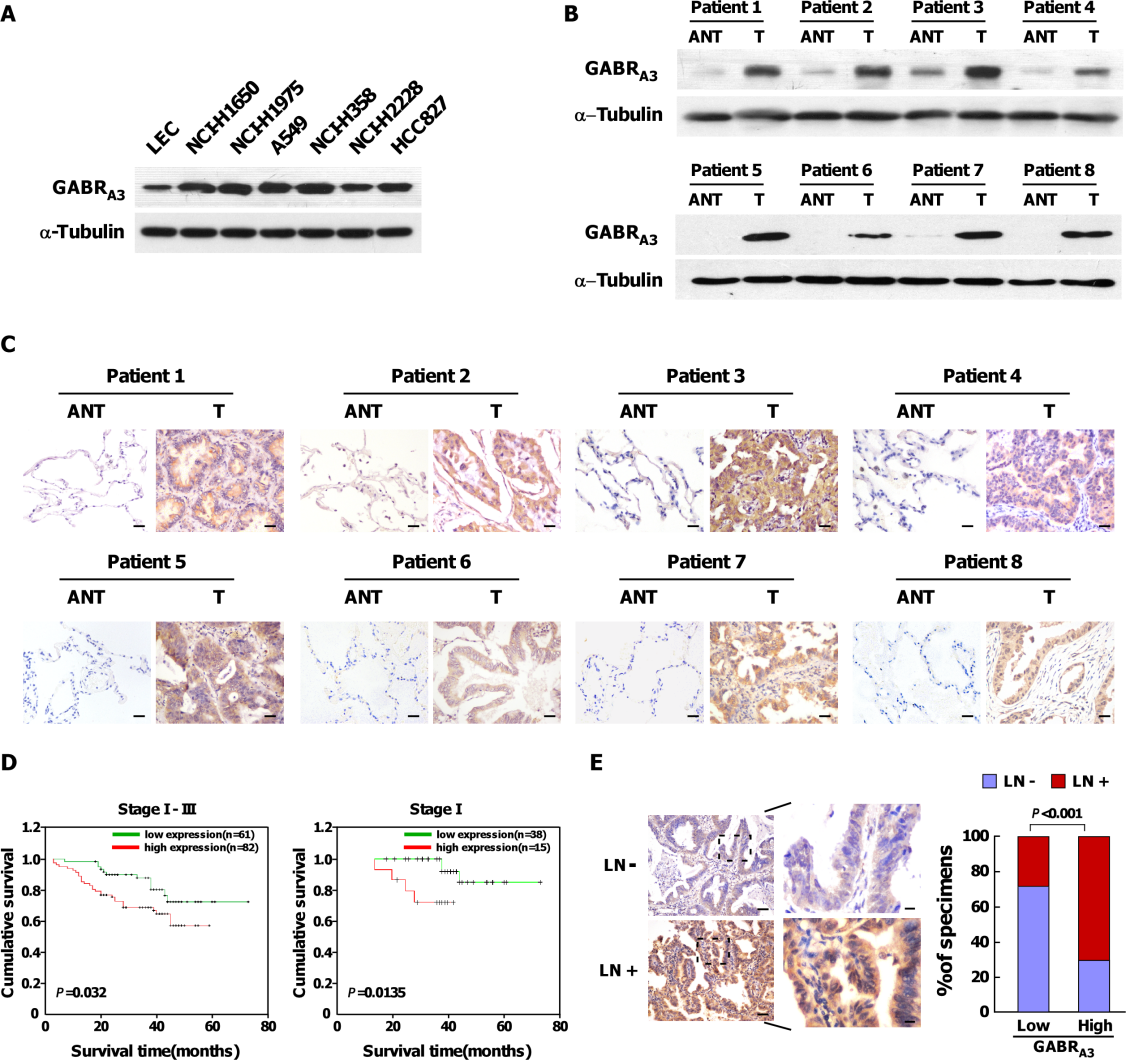
Upregulation of GABR_A3_ correlates with poor prognosis in LUAD (**A**) Western blots showing expression of GABR_A3_ protein in lung epithelial cells and LUAD cells. a-Tubulin was used as a loading control. (**B–C**)Comparison of GABR_A3_ protein expression between primary LUAD tissues (T) and matched adjacent non-tumor tissues (ANT) using Western blot analysis (B) and IHC (C). Scale bars, 50 μm. (**D**). Kaplan-Meier survival curves for LUAD patients showing low and high levels of GABR_A3_ expression. (**E**). Correlation of GABR_A3_ expression patients with lymphatic metastasis and those without metastasis. Left panel: micrographs of two representative cases. Scale bars, 50 mm; insets, 10 mm. Right panel: Chi-square analysis of the relation between low or high GABR_A3_ expression and lymphatic metastasis.

We analyzed the GABA receptor expression profile in 31 pairs of LUAD and their corresponding adjacent non-tumorous lung tissues using RNAseqV2 data sets for LUAD on the TCGA website (https://tcga-data.nci.nih.gov/tcga/). The results showed that the mRNA expression of GABR_A3_ (*t* = 3.477, *P* = 0.002), GABR_A5_ (*t* = 2.121, *P* = 0.042), GABR_D_ (*t* = 3.259, *P* = 0.003), GABR_G2_ (*t* = 2.318, *P* = 0.027), and GABR_Q_ (*t* = 2.219, *P* = 0.034) was higher in LUAD tissues (Supplementary Figures 1 and 2). In addition, expression of GABR_A3_ mRNA in lung squamous cell carcinoma was higher than in matched adjacent non-tumor tissues (*t* = 4.219, *P* = 0.0007; Supplementary Figure 3A). Using real-time quantitative PCR, we confirmed that GABR_A3_ gene expression was upregulated in LUAD cell lines and fresh clinical LUAD tissues as compared to paired non-cancerous tissues (Supplementary Figure 3B and 3C). Correspondingly, levels of GABR_A3_ protein were markedly higher in LUAD cell lines than in normal human lung epithelial cells (Figure 1A), and higher in clinical LUAD tissues than in matched adjacent non-tumor tissues (Figure 1B and 1C). These results demonstrate that GABR_A3_ is overexpressed in human LUAD, at both the protein and mRNA levels.

We also used immunohistochemistry to assess GABR_A3_ protein expression in 143 paraffin-embedded, archived LUAD tissue samples, including 54 cases of grade I (37.7%), 29 cases of grade II (20.3%), and 60 cases of grade III (42%; Supplementary Table 1). Statistical analyses revealed that levels of GABR_A3_ protein correlated significantly with TNM clinical stage (*P* < 0.001), lymph node metastasis status (*P* < 0.001) and patient survival (*P* = 0.042; Supplementary Table 2). Additionally, Kaplan-Meier survival analysis and log-rank test showed that GABR_A3_ overexpression correlated with shorter overall survival in stages I-III (*P* = 0.032) or stage I (*P* = 0.0135; Figure 1D), but not in stage II or III patients. Immunohistochemical detection of GABR_A3_ was adversely associated with survival in univariate analysis (*P* = 0.037; Supplementary Table 3), but was not statistically significant in the multivariate analysis (*P* = 0.554; Supplementary Table 3).

### GABR_A3_ expression correlates with lymphatic metastasis and promotes the invasiveness of LUAD cells *in vitro*

**Figure 2:**
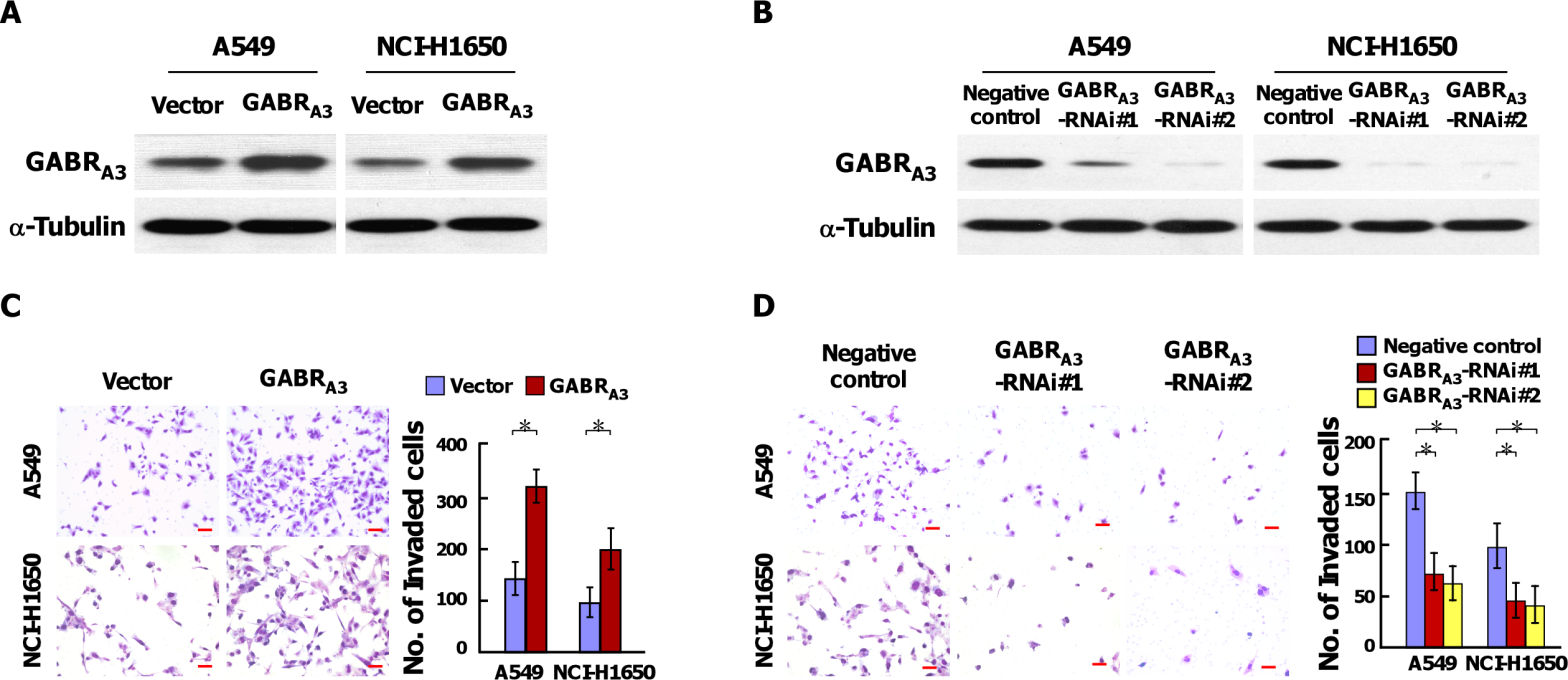
GABR_A3_ promotes the invasiveness of LUAD cells (**A–B**) Western blot analysis of the effect of overexpressing (A) or silencing (B) GABR_A3_ in the A549 and NCI-H1650 cell lines. a-Tubulin was used as a loading control. (**C–D**) Representative micrographs (left panel) and quantification (right panel) of invaded cells in a Transwell matrix penetration assay. Scale bars, 50 mm. Error bars depict the mean ± SD from three independent experiments, *P < 0.05.

As shown in Figure 1E and Supplementary Table 2, levels of GABR_A3_ expression were higher in patients with lymphatic metastasis than without it. This suggests GABR_A3_ may contribute to lymphatic metastasis of LUAD. To test that idea, we established stable GABR_A3_-overexpressing and GABR_A3_ knockdown cells in the A549 and NCI-H1650 lines, respectively (Figure 2A and 2B). Subsequent Transwell matrix penetration assays showed that cells overexpressing GABR_A3_ were more invasive than control cells, whereas silencing GABR_A3_ diminished invasive activity (Figure 2C and 2D). Likewise, knocking down GABR_A3_ expression in our previously established primary Am1010 cells, derived from muscle metastases of a human lung adenocarcinoma (Supplementary Figure 5A) [12], inhibited invasive activity in Transwell assays (Supplementary Figure 5B). These results suggest that GABR_A3_ promotes the invasiveness of LUAD cells.

*In vitro* MTT assay, it demonstrated that the proliferation rate of GABR_A3_-transduced cells, GABR_A3_-silenced cells or control cells has no significant difference (Supplementary Figure 4A and 4B). But, GABR_A3_-transduced cells were more resistant to cisplatin than vector cells, and GABR_A3_-silenced cells were more sensitive to cisplatin than vector cells *in vitro* (Supplementary Figure 4C and 4D).

### GABR_A3_ increases lymph node metastasis *in vivo*

**Figure 3:**
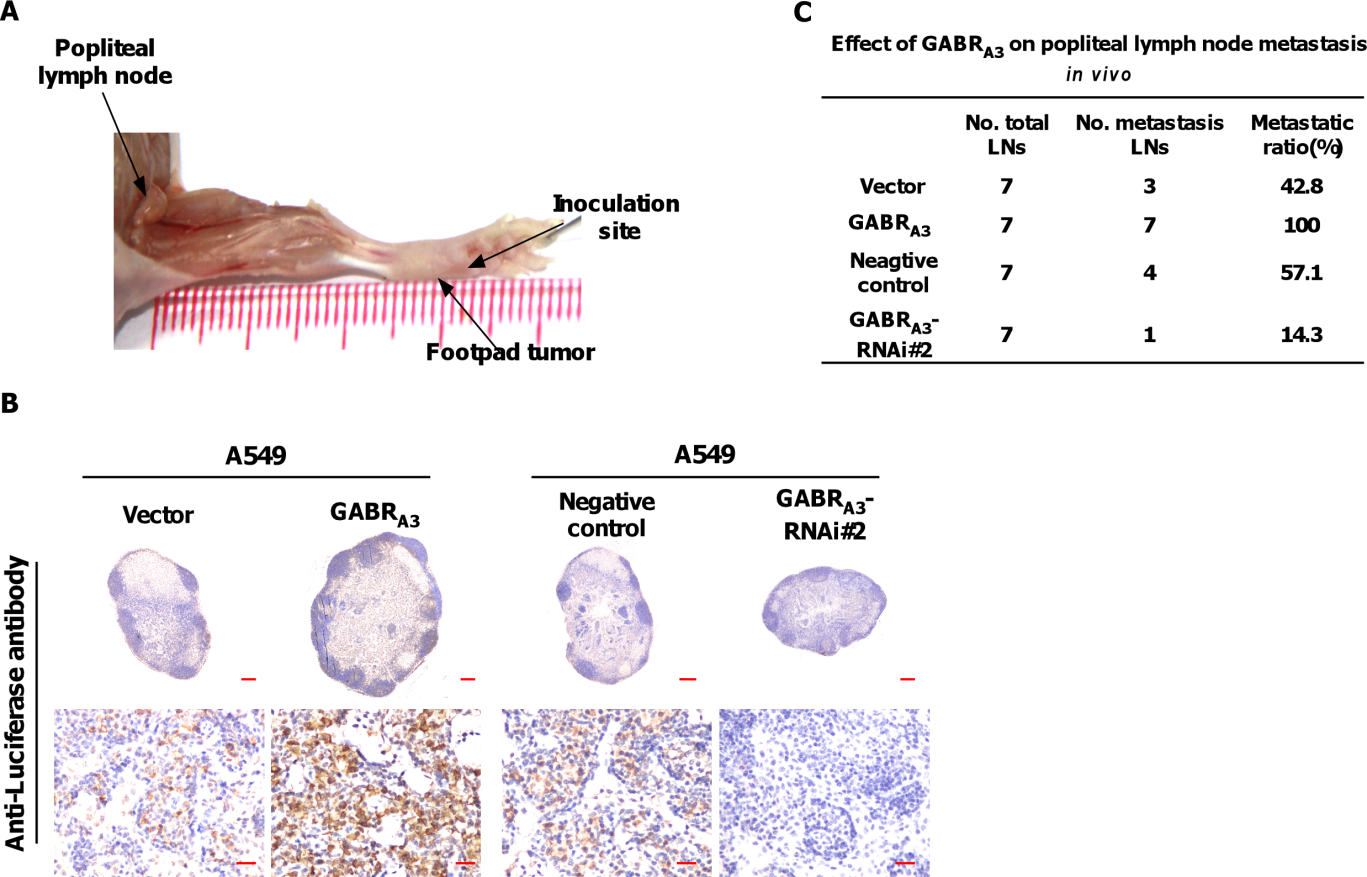
GABR_A3_ promotes lymph node metastasis *in vivo* (**A**) Representative micrographs of the popliteal lymph node metastasis model. The indicated cells stably expressing firefly luciferase were inoculated into the footpads of mice. (**B**) Representative micrographs of popliteal lymph nodes immunostained with anti-luciferase antibody. Scale bars: upper panel, 200 mm; lower panel, 20 mm. (**C**) Ratios of metastatic to total lymph notes in the indicated cells. *P < 0.05.

We inoculated firefly luciferase-expressing, GABR_A3_-overexpressing, GABR_A3_-silenced or control cells into the footpads of nude mice (*n* = 7/group; Figure 3A). When the popliteal lymph nodes were enucleated and fixed 5 weeks later, we found more luciferase-positive tumor cells within lymph nodes from mice injected with GABR_A3_-overexpressing cells than with vector-control cells (Figure 3B). Moreover, lymph nodes from mice injected with GABR_A3_-silenced cells had fewer luciferase-positive tumor cells (Figure 3B). Strikingly, the ratios of metastatic to total nodes were significantly higher in the GABR_A3_-overexpressing group (100% [7/7]) than the vector-control (42.8% [3/7]) or negative control groups (57.1% [4/7]). And only a single metastatic lymph node was detected in the GABR_A3_-silenced groups (Figure 3C). These results indicate that GABR_A3_ promotes lymph node metastasis of LUAD cells *in vivo.*

### GABR_A3_ upregulates MMP-2 and MMP-9 expression in LUAD

**Figure 4:**
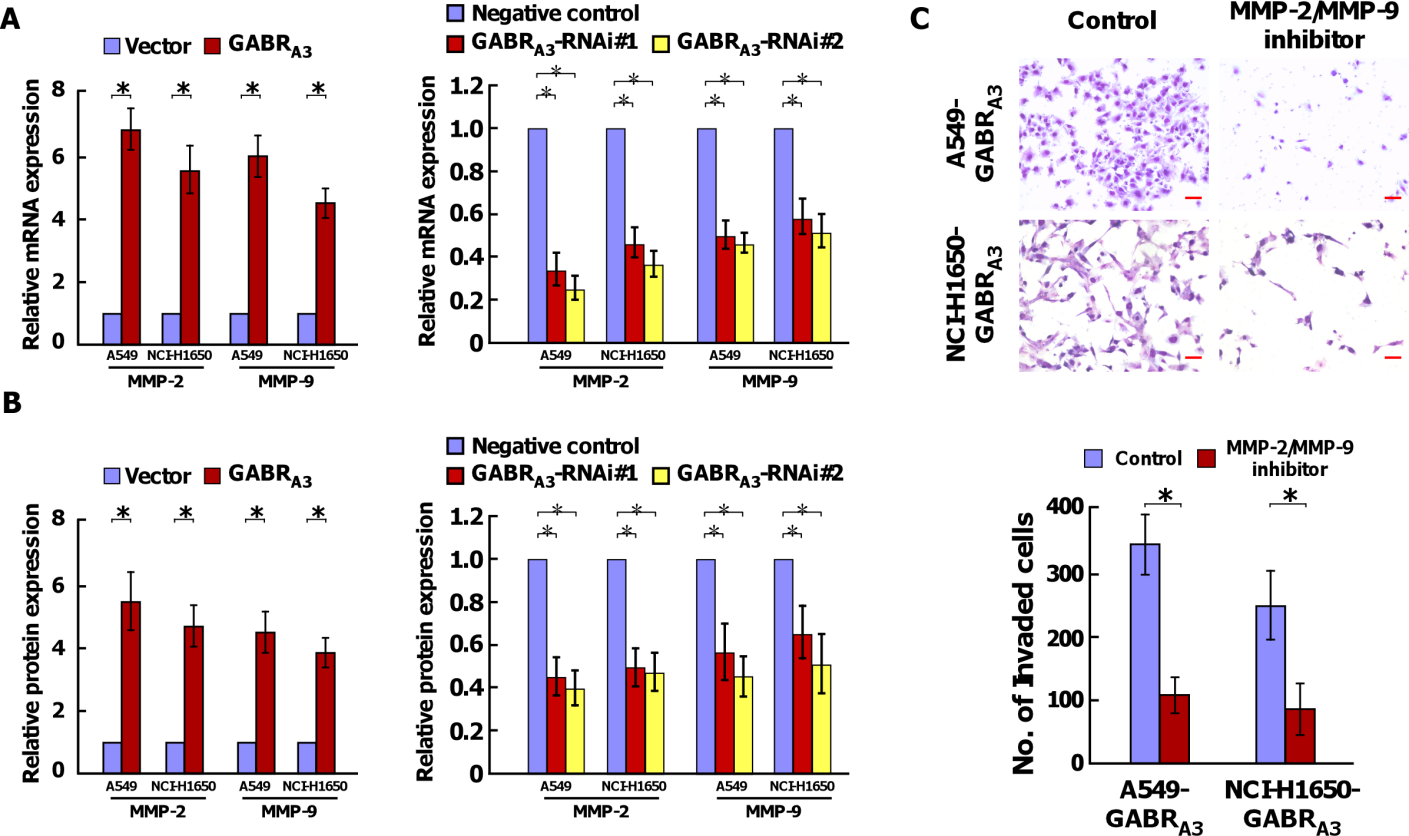
GABR_A3_ induces MMP-2 and MMP-9 expression in LUAD cells (**A**) Real-time PCR analysis of MMP-2 and MMP-9 mRNA expression in the indicated cells. Transcript levels were normalized to GAPDH expression. (**B**) Levels of MMP-2 and MMP-9 protein in supernatants from the indicated cell cultures assessed using ELISAs. (**C**) Representative micrographs (upper panel) and quantification (lower panel) of invaded cells with or without treatment with MMP-2/MMP-9 inhibitor (200 nM). Scale bars, 50 mm. Error bars depict the mean ± SD of three independent experiments, *P < 0.05.

Real-time quantitative PCR analysis showed that levels of MMP-2 and MMP-9 mRNA were increased in GABR_A3_-overexpressing cells and decreased in GABR_A3_-silenced cells (Figure 4A and Supplementary Figure 5C). In addition, ELISAs showed that overexpressing GABR_A3_ also led to increased expression of MMP-2 and MMP-9 proteins, while silencing GABR_A3_ reduced expression of the two enzymes (Figure 4B and Supplementary Figure 5D). Moreover, inhibition of MMP-2 and MMP-9 enzyme activity using MMP-2/MMP-9 Inhibitor V (200 mM) reduced the invasive ability of GABR_A3_-overexpressing LUAD cells (Figure 4C).

**Figure 5:**
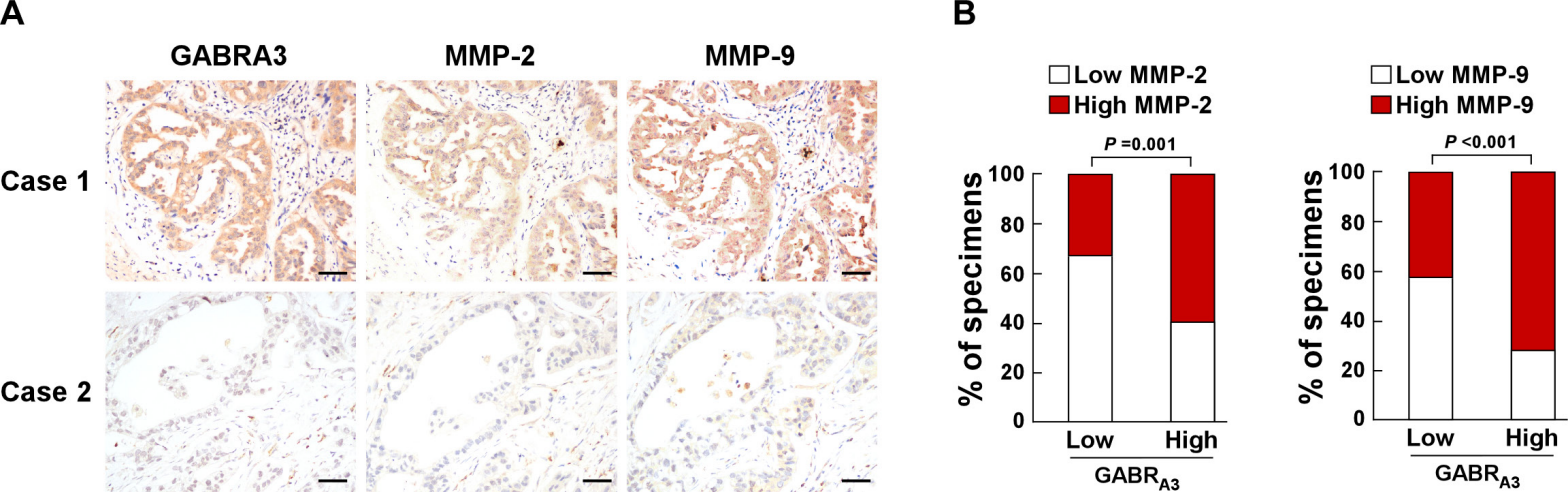
Clinical relevance between GABR_A3_ and MMP-2/MMP-9 expression in human LUAD (**A**) Relation between GABR_A3_ and MMP-2/MMP-9 expression. Shown are representative micrographs of two cases. Scale bars, 50 mm. (**B**) Percentages of LUAD specimens showing low- or high- GABR_A3_ expression in relation to MMP-2/MMP-9 expression.

Immunohistochemical analysis of paraffin-embedded clinical LUAD specimens revealed that MMP-2 and MMP-9 expression was strong in samples with high GABR_A3_ expression, but low in samples with low GABR_A3_ level (Figure 5A). Chi-square analysis showed that GABR_A3_ expression correlated significantly with MMP-2 (*P* = 0.001) and MMP-9 expression (*P* < 0.001; Figure 5B). These results indicate that upregulation of MMP-2 and MMP-9 expression plays a central role in GABR_A3_-mediated LUAD invasiveness.

### JNK/AP-1 signaling pathway strongly contributes to GABR_A3_ induced MMP-2, MMP-9 expression and invasiveness

**Figure 6:**
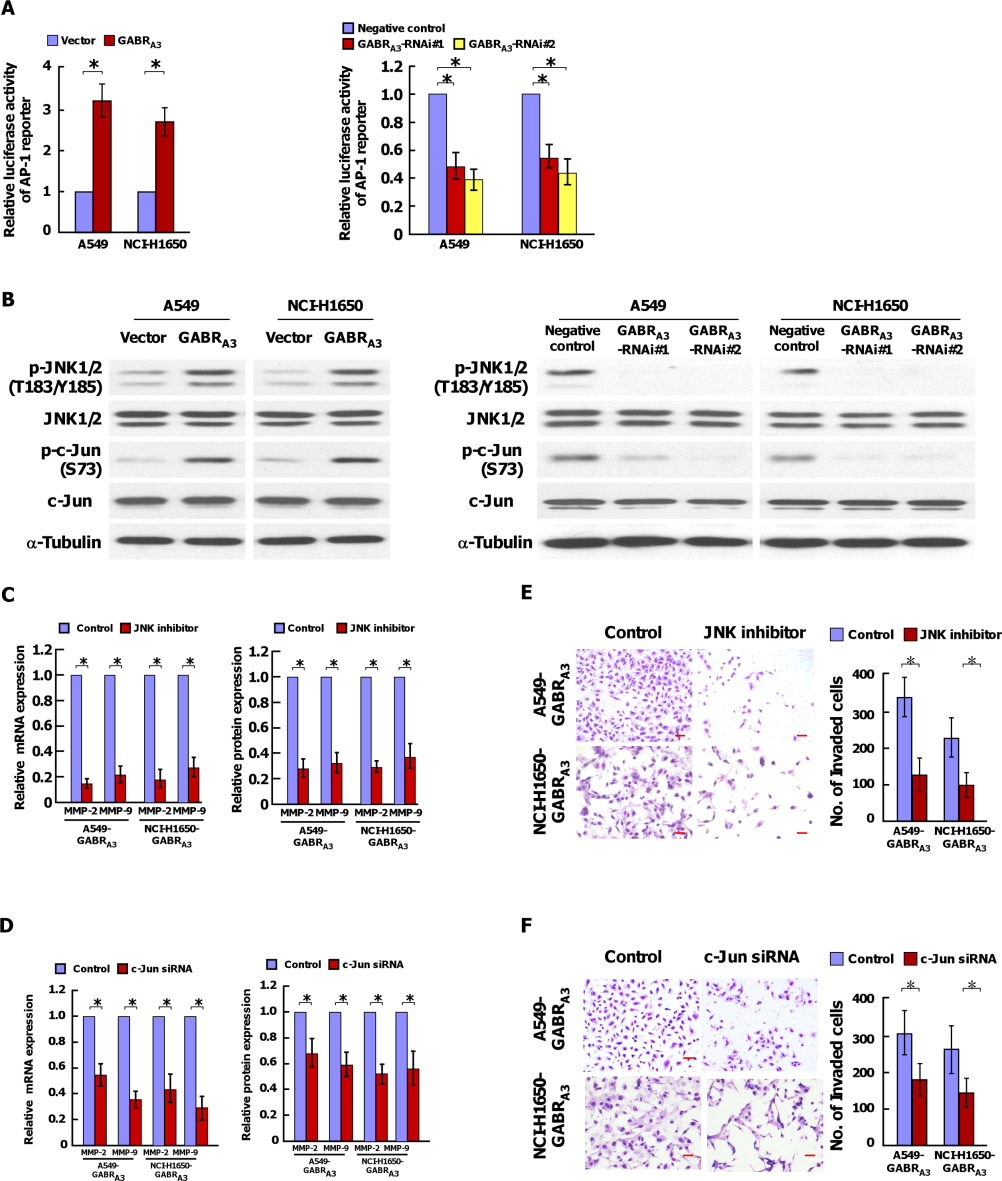
GABR_A3_ activates the JNK/AP-1 signaling pathway (**A**) Transcriptional activities of an AP-1 luciferase reporter plasmid in the indicated cells. (**B**) Western blot analysis of p-JNK1/2(T183/Y185), total JNK1/2, p-c-Jun (S73), and total c-Jun expression in the indicated cells. a-Tubulin was used as a loading control. (**C** and **D**) MMP-2 and MMP-9 mRNA and protein expression in the indicated cells with or without treatment with a JNK inhibitor (150 nM) (C) or c-Jun siRNA (D). E and F. Representative micrographs (left panel) and quantification (right panel) of invaded cells with or without treatment with a JNK inhibitor (**E**) or c-Jun siRNA (**F**). Scale bars, 50 mm. Error bars depict the mean ± SD of three independent experiments, *P < 0.05.

Using dual-luciferase assays, we found that the AP-1-driven luciferase reporter activity was stimulated in GABR_A3_-overexpessing cells, but inhibited in GABR_A3_ silencing cells (Figure 6A). Consistently, the transcriptional levels of cyclin D, cyclin E, p16^INK4^, p21^CIP^, Bcl-2, Bcl-xl, VEGF, downstream genes of AP-1 signaling pathway, were unregulated in GABR_A3_-transduced cells, and decreased in GABR_A3_-silenced cells (Supplementary Figure 6). And, overexpression of GABR_A3_ increased, and silencing GABR_A3_ decreased phosphorylation of JNK1/2 and c-Jun (Figure 6B). Inhibition of the JNK/AP-1 signaling pathway using a JNK inhibitor or silencing c-Jun using targeted siRNA reduced GABR_A3_-driven MMP-2 and MMP-9 expression (Figure 6C and 6D). Furthermore, the stimulatory effect of GABR_A3_ on LUAD invasiveness was reversed by a JNK enzyme inhibitor or c-Jun siRNA, which indicates the importance of the JNK/AP-1 signaling pathway for activation of GABR_A3_-induced LUAD invasion.

## DISCUSSION

Lymph-node metastasis, a crucial step in tumor progression, is a risk factor for disease recurrence and poor prognosis in lung cancer [13, 14]. However, the molecular mechanism underlying lymph node metastasis remains poorly understood. In this study, we report that overexpression of GABR_A3_ in LUAD cells was significantly associated with lymphatic metastasis. Through selective modulation of a JNK/AP-1/MMPs pathway, GABR_A3_ promoted lymphatic metastasis in LUAD cells *in vitro* and *in vivo*.

GABR_A3_ is a subunit of the GABA A receptor [15]. Liu et al. demonstrated that GABR_A3_ is overexpressed in hepatocellular carcinoma and that GABA promoted hepatocellular carcinoma cell proliferation through overexpression of GABR_A3_ [16]. Liu et al. also reported that GABR_A3_ is overexpressed in NSCLC tissues, and that the level of GABR_A3_ expression was associated with the NSCLC grade [11]. Consistent with this observation, we previously showed that higher levels of GABR_A3_ mRNA are associated with disease progression in NSCLC patients [10]. In the present study, our univariate analysis showed that overexpression of GABR_A3_ protein was significantly associated with lymphatic metastasis status (*P* < 0.001) and patient survival (*P* = 0.032) in LUAD. In a multivariate analysis, however, GABR_A3_ expression was not a significant independent prognostic factor (*P* = 0.554). This may be due to insufficient sample size, or it could reflect the relation between GABR_A3_ expression and tumor stage. GABR_A3_ expression and tumor stage, including lymph node involvement, were highly correlated. As such, they would not be significant independent factors when entered into the Cox model at the same time.

MMP-2 and MMP-9 are members of a family of zinc-dependent enzymes that digest and degrade components of the extracellular matrix [17]. This facilitates multiple steps in cancer metastasis, including detachment, invasion, intravasation and extravasation, as well as angiogenesis and lymphangiogenesis [18–23]. Moreover, expression of MMP-2 and MMP-9 is upregulated in many cancer types, including lung cancer, breast cancer, and glioma, and is considered an important prognostic factor [24–26]. Upregulation of MMPs is induced by several oncogenic pathways, the including JNK/AP-1 signaling pathway [27–32]. In the present study, overexpression of GABR_A3_ increased MMP-2 and MMP-9 expression, while silencing GABR_A3_ reduced it. Inhibiting MMP-2/MMP-9 activity abrogated the invasiveness of GABR_A3_-overexpressing cells. In addition, overexpressing GABR_A3_ markedly increased, while silencing GABR_A3_ decreased, the expression of phospho-JNK1/2 (T183/Y185) and phospho-c-Jun (S73) in LUAD cells. What's more the expression of MMP-2/MMP-9 and the invasiveness of GABR_A3_-overexpressing cells was inhibited by the JNK inhibitor SP600125 or by c-Jun siRNA. Thus GABR_A3_-induced lymphatic metastasis in LUAD appears to be via activation of the JNK/AP-1/MMPs axis.

Identifying a key molecule involved in lymphatic metastasis could provide new therapeutic targets for cancer treatment. Our research on GABR_A3_ uncovered the novel molecular mechanism underlying the lymphatic metastasis in LUAD, and may lead to the development of a new therapeutic strategy for the treatment of LUAD.

## MATERIALS AND METHODS

### Cell lines

The A549, NCI-H1650, NCI-H1975, NCI-H358, NCI-H1395 and HCC827 lung adenocarcinoma cell lines were purchased from Shanghai Institutes of Biological Sciences (Shanghai, China) and cultured under the manufacturers suggested conditions. Normal human lung epithelial cells were collected for lung samples as described previously [33].

### Patient information and tissue specimens

A total of 143 paraffin-embedded lung adenocarcinoma specimens that had been histopathologically and clinically diagnosed at the first Affiliated Hospital of Guangzhou Medical University were collected for this study. Follow-up information and cause of death were obtained from a review of telephone follow-ups conducted every 3 months. Twelve (8.39%) patients were lost to follow-up. Clinical information on the patients after complete surgical resection is summarized in Supplementary Table 1. Fresh lung adenocarcinoma tissues and the adjacent normal tissues were obtained from patients who underwent surgical resection with no other anticancer therapies before surgery in the Thoracic Surgery Department of The First Affiliated Hospital of Guangzhou Medical University. Normal lung tissues were obtained from individuals who underwent surgical resection of pulmonary bullae and were confirmed to be free of any prior pathologically detectable conditions. The use of these clinical materials for research purposes was approved by the patients and the Institutional Research Ethics Committee.

### Plasmids, virus production and infection of target cells

The human GABR_A3_ coding sequence was amplified using PCR and cloned into the pMSCV vector. To silence endogenous GABR_A3_, two short hairpin RNA (shRNA; synthesized by Invitrogen) oligonucleotides (GCTGAAGTGGTTTATTCTTGG and GCTCTTTGCCATATTCAATCT) were separately cloned into pSuper-retro-puro vector. A non-targeting shRNA, TTCTCCGAACGTGTCACGT, was used as a negative control. HEK293T cell lines stably expressing GABR_A3_ and GABR_A3_ shRNA were generated through retroviral infection and selected using 0.5 μg/ml puromycin for 10 days. GABR_A3_ levels in the stable cells were detected by Western blotting.

### Real-time quantitative PCR

The relative mRNA levels of selected genes were calculated as 2^−[(Ct of gene) – (Ct of GAPDH)]^ normalized the level of GAPDH mRNA. The sequences of the primers were as follows: for GABR_A3_, 5′-TCGGTCTCTCCAAGTTTG TGC-3′ and 5′-TTCCGTTGTCCACCAATCTGA-3′; for MMP2, 5′-AAGAAGTAGC TGTGACCGCC-3′ and 5′-TTGCTGGAGACAAATTCTGG-3′; for MMP9, 5′-TTGG TCCACCTGGTTCAACT-3′ and 5′-ACGACGTCTTCCA GTACCGA-3′; for GAPDH, 5′-AATCCCATCACCATCT TCCA-3′ and 5′-CCTGCTTCACCACCTTCTTG-3′.

### Western blotting (WB)

Western blotting was performed as previously described [34]. The primary antibodies used were anti-GABR_A3_ (Sigma, Saint Louis, MO), anti-p-JNK1/2, anti-JNK1/2, anti-p-c-Jun and anti-c-Jun (Abcam, Cambridge, MA). The blotted membranes were then stripped and reprobed with an anti-α-tubulin antibody (Sigma, Saint Louis, MO) as a loading control.

### Chemical reagents

MMP-2/MMP-9 inhibitor V was purchased from Calbiochem (San Diego, CA; Cat. No. 444274). JNK inhibitor SP600125 was purchased from R&D Systems (Minneapolis, MN; Cat. No. 1496).

### Immunohistochemistry (IHC)

IHC analysis was performed with 143 paraffin-embedded, archived samples of clinical LUAD tissue as previously described [34]. The intensity of immunostaining was scored separately by two independent pathologists. A staining index (SI) was calculated as the staining intensity score × the proportion of positive tumor cells. Cut-off values for high and low expression of proteins of interest were chosen on the basis of a measure of heterogeneity.

### Popliteal lymph node metastasis model

Cells of interest labeled with firefly luciferase (3 × 10^6^) were inoculated into the footpads of male BALB/c-nu mice (18–20 g), which were then randomly divided into groups (*n* = 7/group) at day 0. All mice were sacrificed after 5 weeks, and all popliteal lymph nodes were enucleated and fixed in formalin. To detect the cancer cells within the popliteal lymph nodes, serial 4.0-μm sections of paraffin-embedded samples were immunohistochemically analyzed using an anti-luciferase antibody (Abcam, Cambridge, MA).

### ELISA

The concentrations of MMP-2 and MMP-9 in medium conditioned by cells of interest were determined using commercially available MMP-2 and MMP-9 ELISA Kits (Abcam, Cambridge, MA) according to the manufacturer's instructions.

### Luciferase assay

AP-1 luciferase reporter plasmids plus pRL-TK renilla plasmid (Promega, Madison, WI) were transfected into cells using Lipofectamine 2000 reagent (Invitrogen, Carlsbad, CA) according to the manufacturer's instructions. Twenty-four hours after transfection, the luciferase and renilla signals were measured using a Dual Luciferase Reporter Assay Kit (Promega, Madison, WI).

### Cell invasion assay

The indicated cells (2 × 10^4^) in serum-free DMEM were plated on the top surface of polycarbonate membranes (coated with Matrigel) in Transwell chambers (Costar, Corning Inc., NY). After incubation for 22 h at 37°C, invaded cells on the lower membrane surface were fixed, stained, photographed and counted under a microscope (10 random fields per well, 100× magnification).

### Statistical analysis

All statistical analyses were performed using SPSS 13.0 statistical software. Paired-samples *t*-tests were used to assess differences in gene expression between LUAD and adjacent non-tumorous lung tissues. The relationship between GABR_A3_ expression and the clinicopathological characteristics was assessed using the chi-square test. Survival curves were plotted using the Kaplan-Meier method and compared using the log-rank test. *P* < 0.05 was considered statistically significant in all experiments.

## SUPPLEMENTARY MATERIALS FIGURES AND TABLES


