# Supplementary material for: GABRA3 promotes lymphatic metastasis in lung adenocarcinoma by mediating upregulation of matrix metalloproteinases
Source: Oncotarget. 2016 Apr 11;7(22):32341–50. doi: 10.18632/oncotarget.8700 (PMC5078017; doi:10.18632/oncotarget.8700)
Supplement: Supplementary file 1 [file oncotarget-07-32341-s001.pdf]

GABR$_{A3}$ promotes lymphatic metastasis in lung adenocarcinoma by mediating upregulation of matrix metalloproteinases

Supplementary Materials

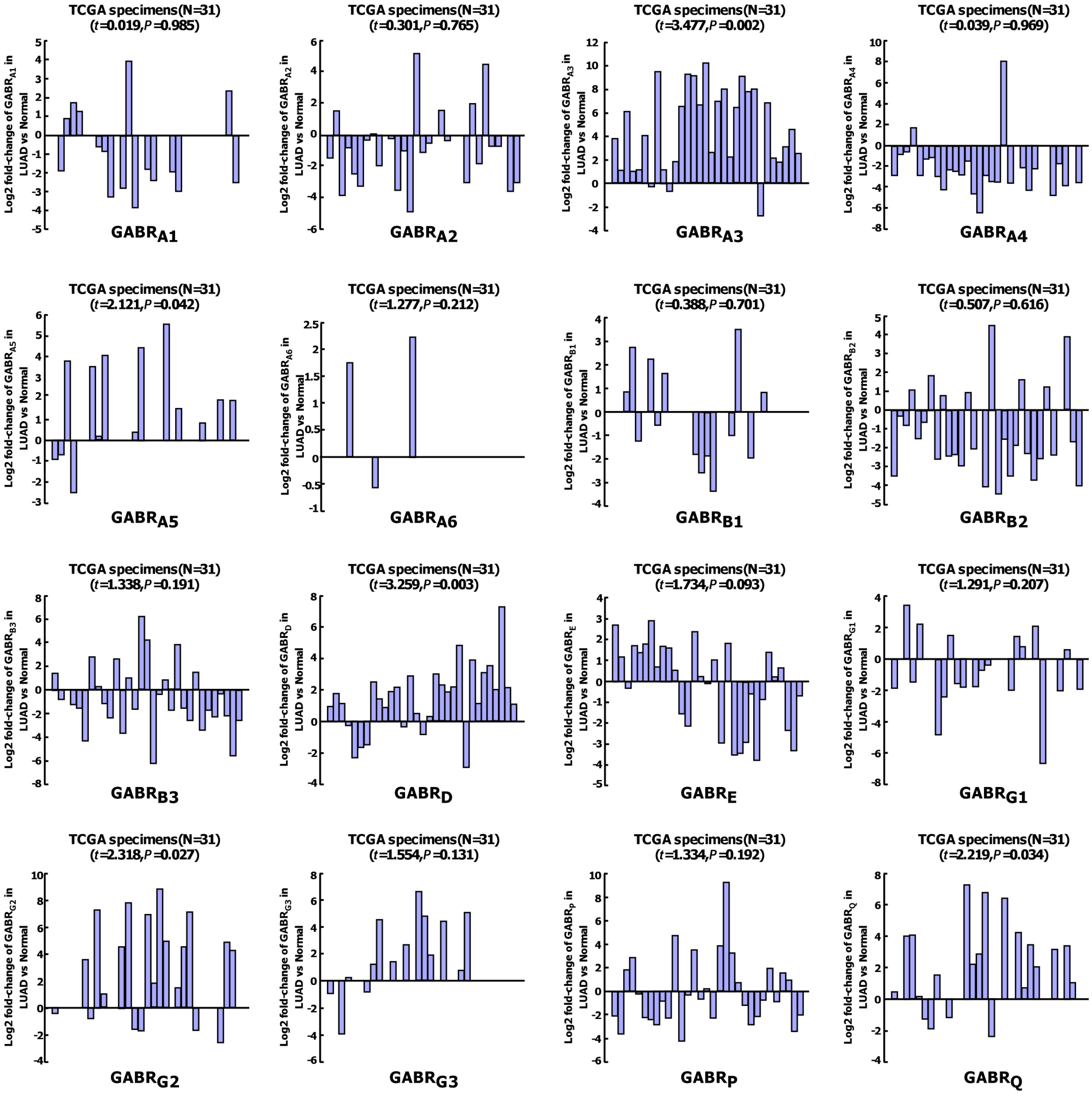

Supplementary Figure S1: Expression of the subunits of GABA A receptor in 31 paired LUAD tissues versus non-tumorous lung tissues using RNAseqV2 data sets on TCGA website.

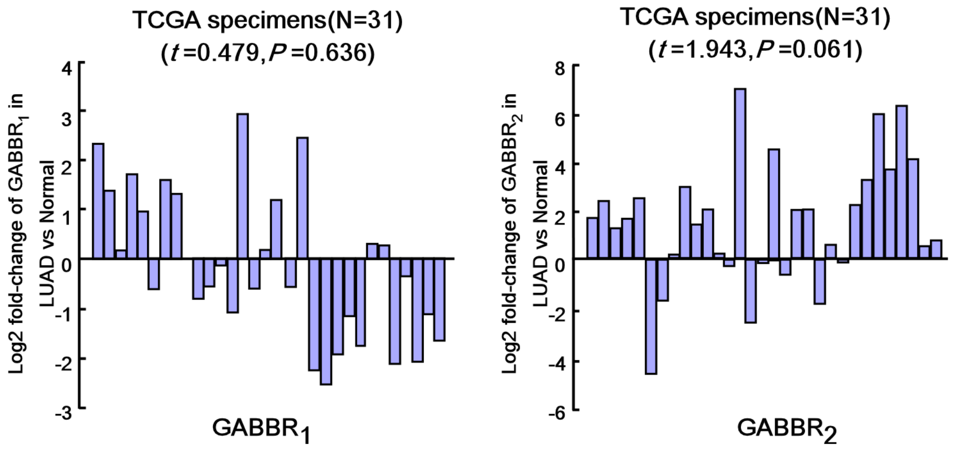

Supplementary Figure S2: Expression of the subunits of GABA B receptor in 31 paired LUAD tissues versus non-tumorous lung tissues using RNAseqV2 data sets on TCGA website.

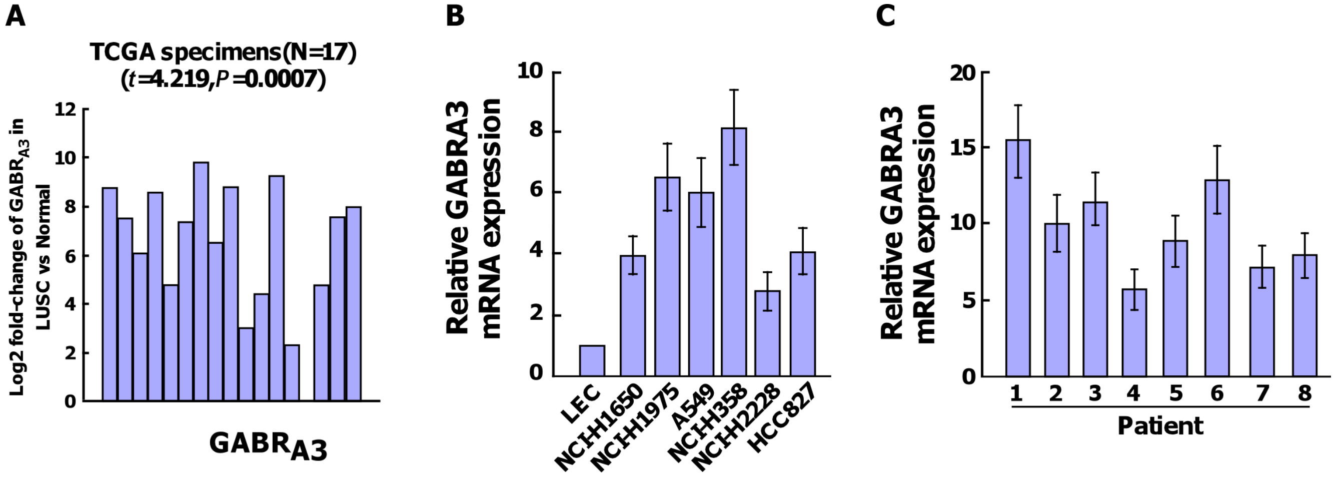

Supplementary Figure S3: GABR$_{A3}$ mRNA expression is upregulated in nonsmall-cell lung cancer. (**A**) Expression analysis of GABR$_{A3}$ in 17 paired LUSC tissues versus non-cancerous lung tissues using RNAseqV2 data sets on TCGA website. (**B**) Real-time PCR analysis of GABR$_{A3}$ mRNA expression in 6 indicated LUAD cell lines and normal human lung epithelial cells. (**C**) Real-time PCR analysis of GABR$_{A3}$ mRNA expression in clinical LUAD tissues compared with paired non-cancerous tissues. Transcript levels were normalized to GAPDH expression. Error bars represent the mean ± SD from three independent experiments, $^{*}P < 0.05$.

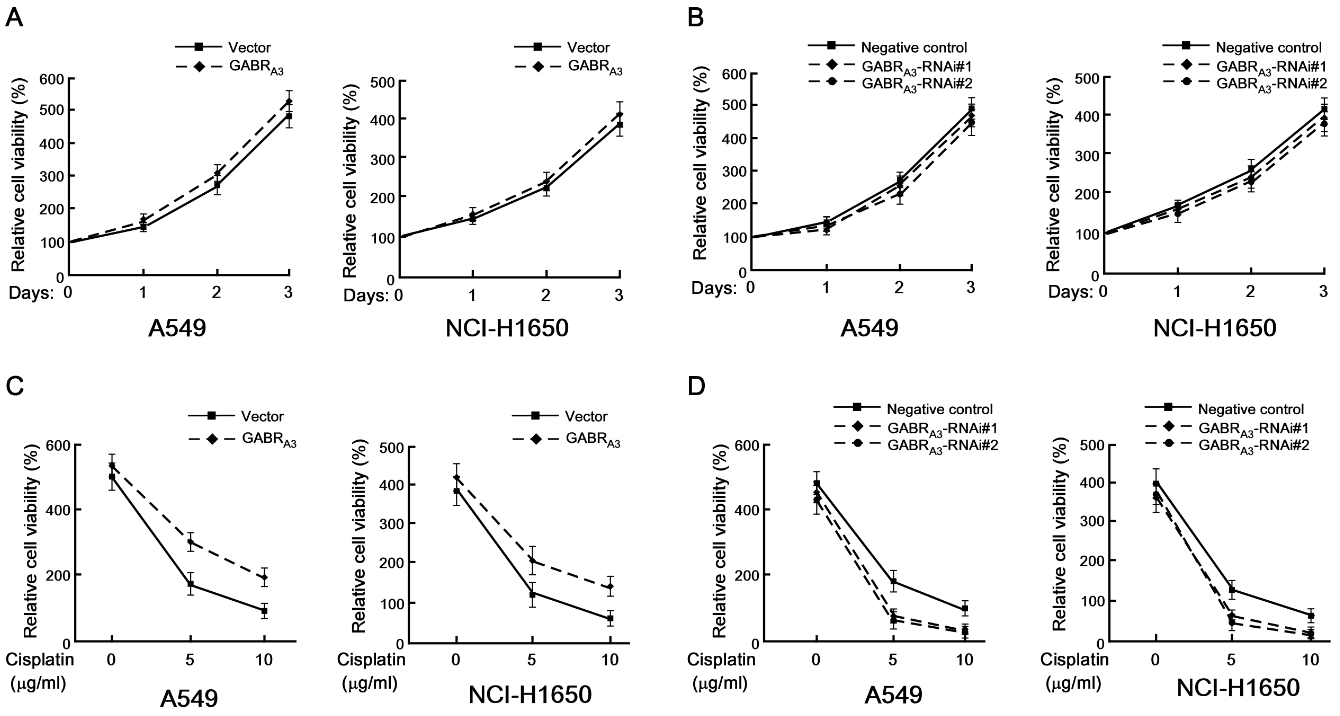

Supplementary Figure S4: Effect of GABR$_{A3}$ on cell proliferation and apoptosis. (**A** and **B**) Effects of overexpression (A) or silencing (B) effect of GABR$_{A3}$ on the proliferation of the indicated lung cancer cell lines, as analyzed by the MTT assay. C and D. Effects of overexpression (**C**) or silencing (**D**) effect of GABR$_{A3}$ on the chemoresistance of the indicated lung cancer cell lines, as analyzed by the cisplatin treatment assay.

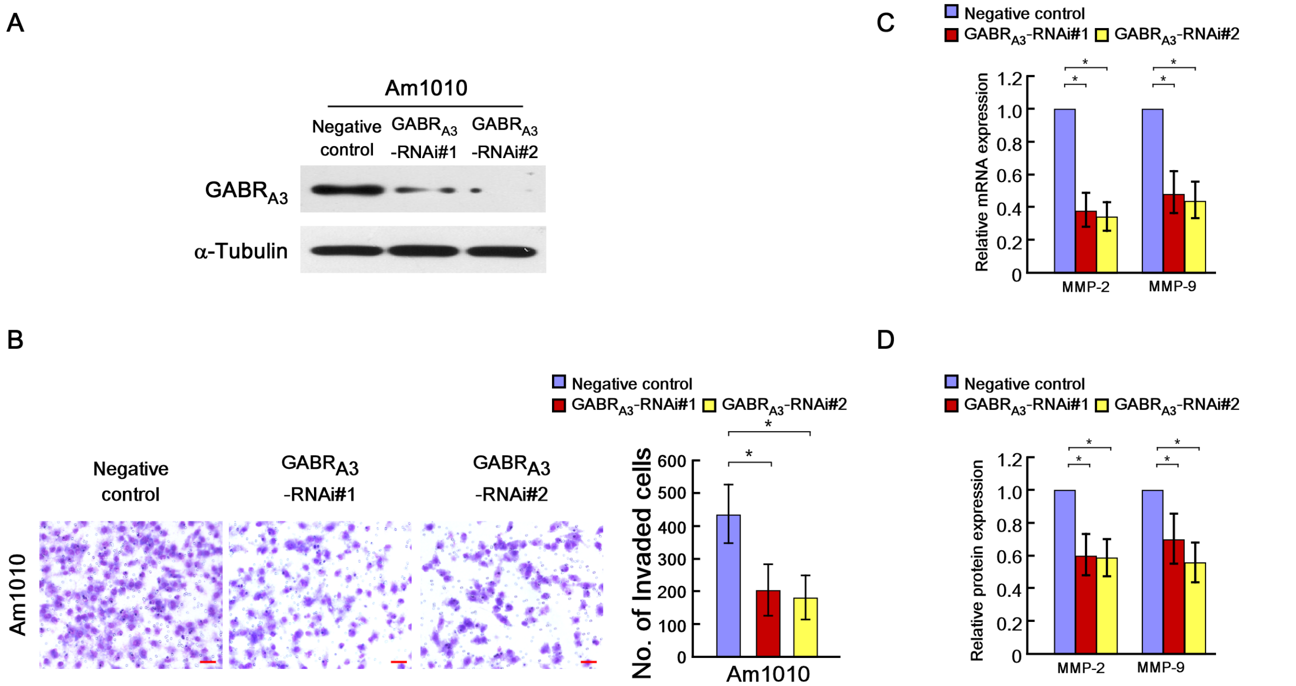

Supplementary Figure S5: Silencing GABR$_{A3}$ decreases MMP2 and MMP-9 expression and inhibits the invasiveness of lung cancer primary cell Am1010. (**A**) Western blot analysis of the effect of silencing GABR$_{A3}$ in Am1010 cell. α-Tubulin was used as a loading control. (**B**) Representative micrographs (left panel) and quantification (right panel) of indicated invaded cells by the transwell matrix penetration assay. Scale bars: 50 μm. (**C**) Real-time PCR analysis of MMP-2 and MMP-9 mRNA expression in the indicated cells. Transcript levels were normalized by GAPDH expression. (**D**) MMP-2 and MMP-9 protein levels in the supernatants of indicated cell cultures were assessed using ELISAs. Error bars represent the mean ± SD from three independent experiments, *$P < 0.05$.

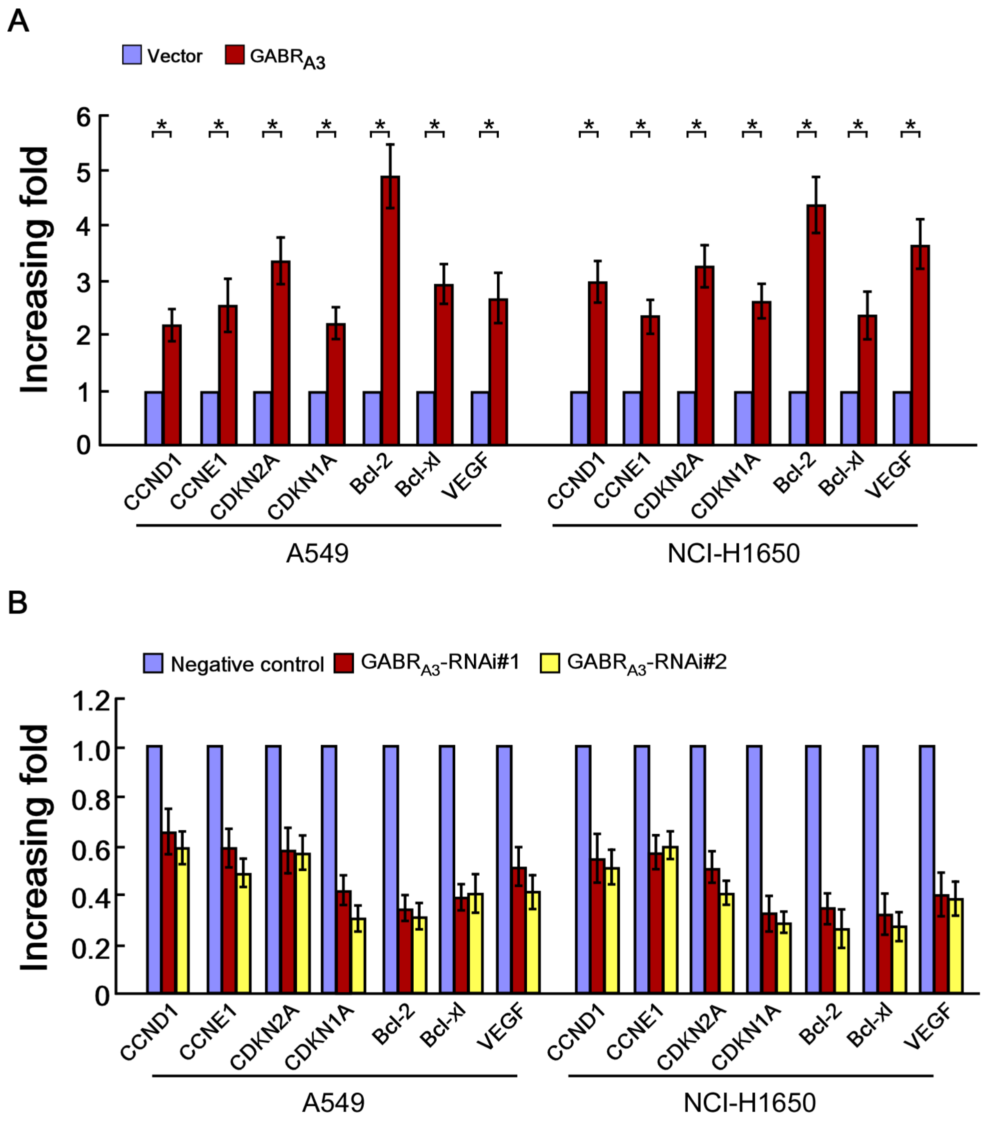

Supplementary Figure S6: GABRA3 induces AP-1-dependent downstream genes expression in LUAD cells. (**A** and **B**) Real-time PCR analysis of *CCND1, CCNE1, CDKN2A,CDKN1A, Bcl-2, Bcl-xl* and *VEGF*, the AP-1 downstream genes, in vector- or $GABR_{A3}$-transduced cells(A), or negative control- or $GABR_{A3}$-silenced cells (B). Transcript levels were normalized to GAPDH expression. Error bars represent the mean ± SD of three independent experiments. **P* < 0.05.

Supplementary Table S1: Clinicopathological characteristics of studied patients and expression of $GABR_{A3}$ in lung adenocacinoma

Factor	No	(%)
**Gender**		
Male	75	52.4
Female	68	47.6
**Age (years)**		
< 60	69	48.3
≥ 60	74	51.7
**Clinical stage**		
I	54	37.7
II	29	20.3
III	60	42.0
**N classification**		
Yes	77	53.8
No	66	46.2
**Patient survival**		
Alive	102	71.3
Deceased	41	28.7
**Expression of GABRA3**		
Low expression	61	42.7
High expression	82	57.3

Supplementary Table S2: Correlation between the clinicopathological features and expression of $GABR_{A3}$

Patient characteristics		GABRA3 expression		*P*-value
		Low	High	
**Gender**	Male	34	41	0.504
	Female	27	41	
**Age (years)**	< 60	27	42	0.499
	≥ 60	34	40	
**Clinical stage**	I	38	16	< 0.001
	II	12	17	
	III	11	49	
**N classification**	Yes	17	60	< 0.001
	No	44	22	
**Survival**	Alive	49	53	0.042
	Deceased	12	29	

Supplementary Table S3: Univariate and multivariate analysis of different prognostic parameters in patients with lung adenocacinoma by Cox-regression analysis

Variables	No. patients	Univariate analysis		Multivariate analysis	
		HR *P*-value	Hazard Ration (95% CI)	HR *P*-value	Hazard Ration (95% CI)
**Gender**					
< 60	69	-	1	-	-
≥ 60	74	0.716	1.121 (0.605–2.077)	-	-
**Gender**				-	-
Male	75	-	1	-	-
Female	68	0.242	0.688 (0.367–1.288)	-	-
**Clinical stage**					
I	54	-	1	-	-
II	29	0.239	1.842 (0.666–5.09)	0.667	1.701 (0.594–4.866)
III	60	0.003	3.28 (1.483–7.252)	0.023	2.869 (1.159–7.101)
**$GABR_{A3}$ expression**					
Low	61	-	1	-	-
High	82	0.037	2.046 (1.044–4.013)	0.554	1.262 (0.584–2.728)

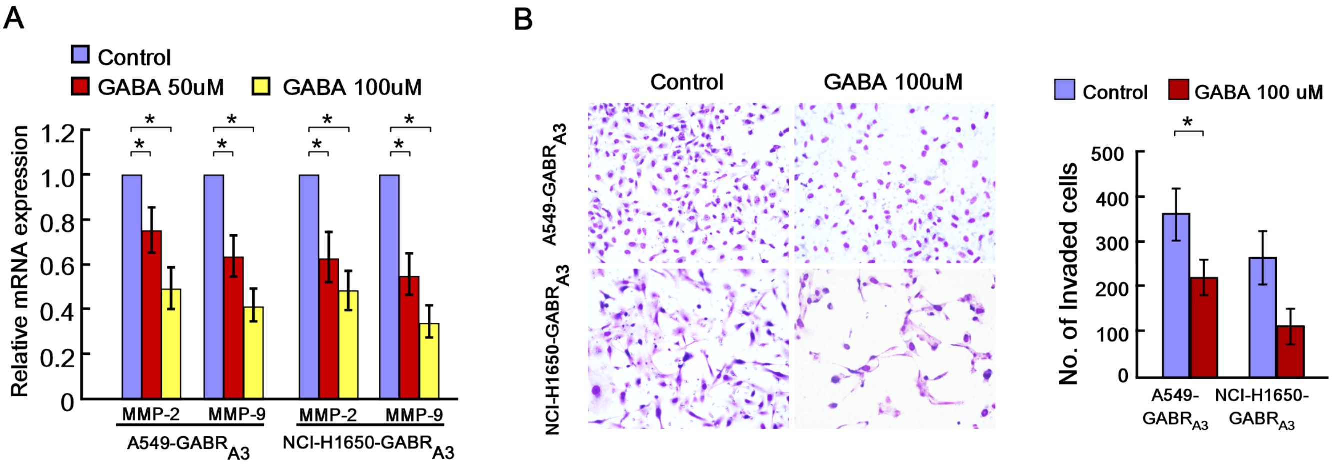

Supplementary Figure for the editors reviewers
